# Effectiveness and safety of electroacupuncture in female overactive bladder: a randomized controlled trial investigating sacral and tibial nerve modulation

**DOI:** 10.3389/fmed.2025.1579276

**Published:** 2025-09-16

**Authors:** Zilong Tan, Mengdi Ding, Junru Li, Ran Luo, Jianwu Shen

**Affiliations:** ^1^Department of Urology, Xiyuan Hospital, China Academy of Chinese Medical Sciences, Beijing, China; ^2^Department of Internal Medicine, Qinghai Provincial Hospital of Traditional Chinese Medicine, Xining, China; ^3^Department of Gynecology, Xiyuan Hospital, China Academy of Chinese Medical Sciences, Beijing, China; ^4^Department of Urology, Qinghai Provincial Hospital of Traditional Chinese Medicine, Xining, China

**Keywords:** electroacupuncture, overactive bladder, sacral neuromodulation, tibial neuromodulation, urinary cytokines

## Abstract

**Background:**

Overactive bladder (OAB) is a common condition in women, affecting quality of life with symptoms like urgency, frequency, and nocturia. Current treatments, such as antimuscarinic drugs, have side effects that limit their effectiveness. Electroacupuncture (EA) shows promise as an alternative, but its mechanisms and effectiveness for OAB are not fully understood.

**Objective:**

This blinded, randomized controlled trial aimed to evaluate the efficacy and safety of electroacupuncture as a therapeutic intervention for female patients with OAB and to explore potential mechanisms involving the sacral and posterior tibial nerves.

**Methods:**

Sixty-eight female OAB patients were stratified and randomized into two groups. One group received EA treatment at BL33 and SP6 acupoints three times weekly for 4 weeks, while the control group received tolterodine, a standard antimuscarinic medication. Outcome measures included urgency symptoms, Overactive Bladder Symptom Score (OABSS), and quality of life at 2 and 4 weeks post-treatment, as well as at a 3-month follow-up. Safety and acceptance of EA were also assessed. Additionally, urinary cytokine levels were analyzed to investigate the neurobiological impact of the treatments.

**Results:**

No significant baseline differences were observed between the groups. At 2 weeks, EA significantly improved quality of life scores (*p* = 0.002), and by 4 weeks, both groups showed improvements in urgency symptoms and quality of life (*p* < 0.05), with no significant difference in OABSS (*p* = 0.081). The EA group demonstrated a significantly higher overall effective rate (88.6%) compared to the medication group (48.5%) (*p* = 0.002). Safety assessments indicated high acceptance and minimal discomfort with EA, while post-treatment urinary cytokine analysis revealed significant changes in BDNF levels, suggesting a neurobiological effect of EA.

**Conclusion:**

Electroacupuncture at BL33 and SP6 is a promising, well-tolerated, and effective intervention for OAB, supporting its integration into treatment paradigms. Further research is needed to optimize its clinical application.

**Clinical trial registration:**

clinicaltrials.gov, identifier ChiCTR-1900021372.

## 1 Introduction

Overactive bladder (OAB) is a common functional urological disorder characterized by urinary urgency, often accompanied by increased frequency and nocturia, with or without urgency incontinence. According to the International Continence Society (ICS), OAB is defined as a symptom-based syndrome in the absence of urinary tract infection or other obvious pathology (1, 2). Globally, the condition affects approximately 12–21% of adults, with a higher prevalence among women (22.1%) than men (19.5%), and incidence increases substantially with age (3, 4). Despite its high prevalence and significant impact on health-related quality of life (HRQoL), underdiagnosis and limited healthcare-seeking behavior remain common (5). Current pharmacotherapies, particularly muscarinic receptor antagonists, are associated with adverse effects such as dry mouth, constipation, and cognitive decline, leading to poor long-term adherence (6–9). These limitations underscore the need for safer and more tolerable therapeutic alternatives.

Neuromodulation has emerged as a key non-pharmacological approach in the management of refractory OAB. Sacral neuromodulation (SNM) delivers electrical impulses to the sacral nerve roots to modulate abnormal reflex pathways involved in bladder function and has shown proven efficacy (10–12). However, the high cost, invasiveness, and potential for diminishing efficacy over time limit its widespread use (13–15). Posterior tibial nerve stimulation (PTNS) offers a less invasive alternative, stimulating afferent fibers of the tibial nerve via surface or needle electrodes, but often requires multiple sessions with uncertain long-term outcomes (16, 17).

Electroacupuncture (EA), a therapy rooted in traditional Chinese medicine and increasingly recognized in modern neuromodulation frameworks, may offer a promising complementary strategy. The BL33 (Zhongliao) and SP6 (Sanyinjiao) acupoints are anatomically aligned with sacral and tibial nerve trajectories, allowing EA to mimic dual-site neuromodulation through minimally invasive means (18–21). This study employed a randomized, evaluator-blinded, positive-controlled design to evaluate the clinical efficacy and safety of EA in female patients with OAB. The EA group received stimulation at bilateral BL33 and SP6, simulating sacral and tibial nerve pathways, while the control group received tolterodine tartrate extended-release tablets. The primary objective was to assess whether EA can effectively alleviate OAB symptoms, improve patient-reported outcomes, and provide mechanistic insight into its neuromodulatory action through urinary cytokine analysis.

## 2 Methods

### 2.1 Design

This study was a single-center, assessor-blinded, parallel-group randomized controlled trial (RCT). Participants were randomized into two groups: an EA group and a medication control group. Outcome measurements were assessed at baseline, 2 weeks after the start of treatment, 4 weeks after the start of treatment, and at a 3 months follow-up. The trial adhered to the Consolidated Standards of Reporting Trials (CONSORT) (22) and the Revised STandards for Reporting Interventions in Clinical Trials of Acupuncture (STRICTA) guidelines (23).

The trial was conducted at the Xiyuan Hospital, China Academy of Chinese Medical Sciences, with approval from the hospital’s ethics committee (Approval number: 2018XLA070-3). A qualified clinical trial monitor was involved to ensure the quality of the trial, providing oversight and recommendations for any issues during the trial process. The trial was registered in the Chinese Clinical Trial Registry (ChiCTR-1900021372).

### 2.2 Participants

Participants were recruited from Xiyuan Hospital, China Academy of Traditional Chinese Medicine, between January 2019 and February 2022. Recruitment included outpatient clinics and online advertisements. All participants provided written informed consent before enrollment.

Participants were included if they met the following criteria: (1) female patients aged between 18 and 60 years; (2) met the diagnostic criteria for overactive bladder (OAB), as defined by the International Continence Society; (3) had not used any Chinese or Western medications for OAB within the past week; and (4) voluntarily signed the informed consent form and agreed to participate in the trial.

Participants were excluded if they met any of the following conditions: (1) presence of urinary tract infection or lower urinary tract obstruction; (2) diagnosis of neurogenic bladder; (3) urethral dysfunction; (4) urinary retention or post-void residual volume >50 mL; (5) severe myasthenia gravis; (6) history of ulcerative colitis or narrow-angle glaucoma; (7) known allergy to tolterodine tartrate tablets; (8) deemed by the investigators to be unable to comply with the study procedures; (9) non-compliance with medication, incomplete data, or other conditions that affect efficacy evaluation; (10) pregnant or breastfeeding; and (11) use of a cardiac pacemaker, metal allergy, or severe needle phobia.

Subjects were excluded from efficacy analysis under the following circumstances: (1) severe protocol violations of the inclusion or exclusion criteria; that is, participants who should not have been randomized; (2) failure to receive any treatment after enrollment; and (3) premature discontinuation due to adverse drug reactions (these cases were excluded from efficacy analysis but included in safety evaluation).

Participants were withdrawn from the trial under the following conditions: (1) withdrawal as decided by the investigators; (2) worsening of the condition during the trial, necessitating termination of participation as judged by the physician; and (3) poor medication adherence (compliance <80%), or self-initiated changes in medication regimen, including addition of medications prohibited by the protocol.

Participants who deviated from the inclusion or exclusion criteria, failed to receive treatment, or discontinued treatment due to adverse drug reactions were excluded from the efficacy evaluation but included in adverse event reporting.

### 2.3 Sample size and randomization

Based on the anticipated efficacy rates derived from preliminary studies and literature reviews (24–26), with effectiveness rates estimated between 58 and 68% for one group and 81 and 90% for the other, a sample size calculation was performed. Assuming a significance level (*α*) of 0.05 and a statistical power (1-*β*) of 0.90, and accounting for an expected dropout rate of 20%, the minimum required sample size for each group was calculated to be 34 participants. Thus, the total sample size for both groups combined was 68 participants, ensuring the study was adequately powered to detect clinically significant differences in outcomes. Stratified blocked randomization was employed to allocate participants into two groups (EA group and medication group) based on the severity of baseline OAB symptoms (mild–moderate and severe). A computer-generated random number table was used to assign participants in a 1:1 ratio. The randomization process was concealed, and the allocation sequence was held by a single administrator. This administrator was the only person aware of group assignments until the intervention began. The allocation was concealed from participants, healthcare providers, and other researchers involved in the study to ensure blinding.

While the treatment group assignment was disclosed to the acupuncturists at the time of treatment initiation, outcome assessors and statisticians remained blinded throughout the study to ensure unbiased evaluation of outcomes.

### 2.4 Electroacupuncture group

**Figure 1:**
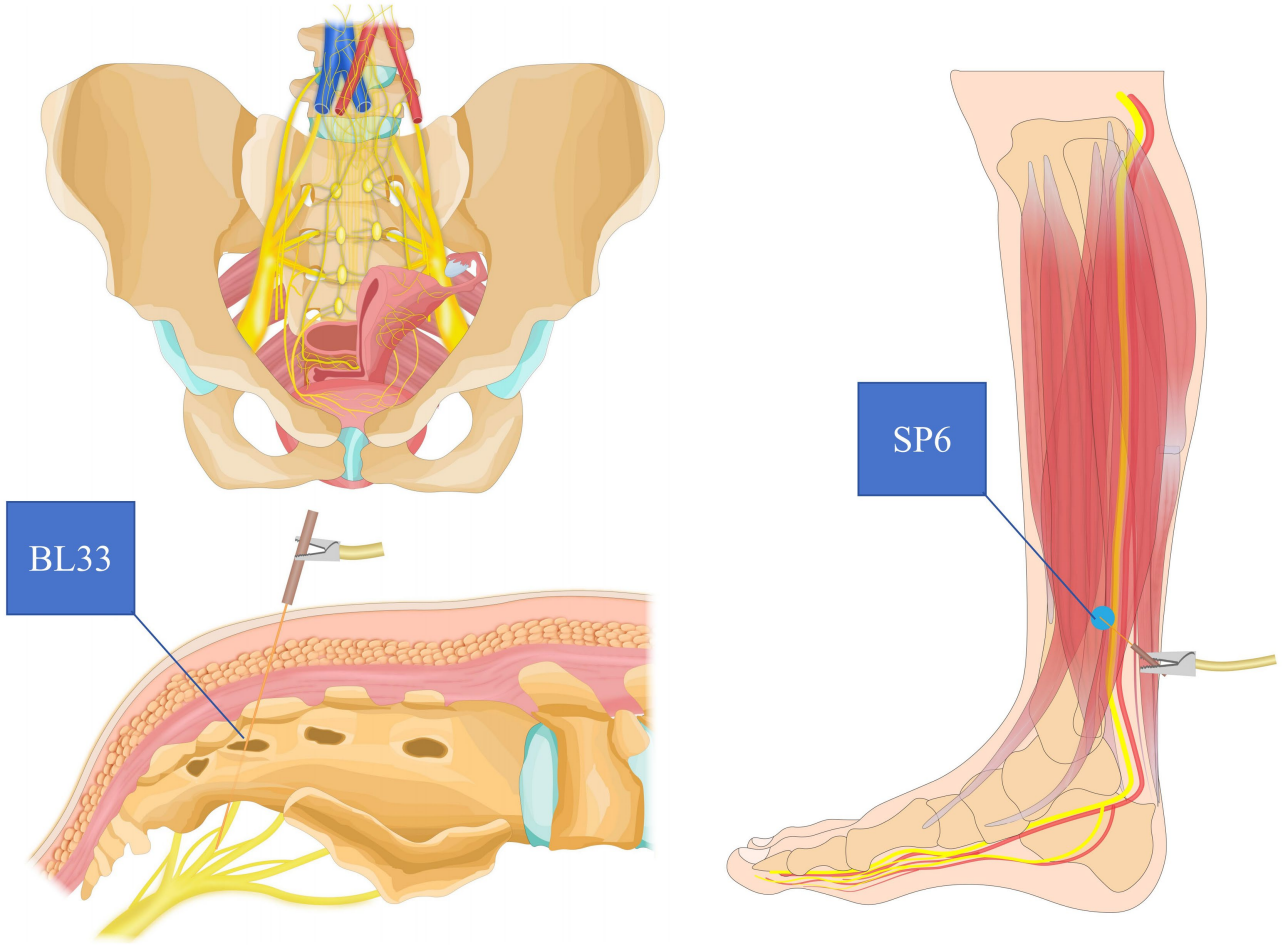
Electroacupuncture stimulation of the sacral nerve S3 via BL33 and the posterior tibial nerve via SP6.

All treatments were performed by trained acupuncturists with at least 5 years of clinical acupuncture experience. Participants in the EA group received real acupuncture with electrostimulation at the BL33 (Baliao) and SP6 (Sanyinjiao) acupoints. Acupuncturists performed needle manipulation, including lifting, thrusting, and rotating the needles to achieve the de qi sensation (a characteristic needle sensation indicating proper stimulation). A 3-inch (0.30 × 75 mm) filiform needle was used for BL33, inserted at a depth of 50–60 mm, while a 2-inch (0.30 × 50 mm) needle was used for SP6, inserted to a depth of 30–40 mm. BL33 Acupoint: Located approximately 1 cm lateral to the third sacral foramen. The needle was inserted at a 30–45° angle downward. SP6 Acupoint: Located on the medial aspect of the lower leg. The needle was inserted perpendicularly (Figure 1). Once the de qi sensation was achieved, electrodes were connected to the needles at BL33 and SP6, and electroacupuncture stimulation was applied using a sparse-dense wave with a frequency of 50 Hz (SDZ-V Huatuo Electronic Acupuncture Instrument, Suzhou Medical Supplies Factory Co., Ltd.). The electrical current intensity was adjusted from 1 mA to 5 mA, based on the participant’s tolerance. Each EA session lasted for 30 min (27, 28).

### 2.5 Control group

Participants in the control group received Tolterodine tartrate extended-release tablets (4 mg/day) manufactured by Nanjing Meirui Pharmaceutical Co., Ltd. (National Drug Approval H20000602). The treatment lasted for 4 weeks, and participants were instructed not to take any additional medications for overactive bladder during this period.

### 2.6 Outcome measures

Baseline and clinical data collection: At the start of the study, baseline demographic and clinical data were gathered to ensure consistency among participants. The primary outcome, assessed with the Overactive Bladder Symptom Score (OABSS) (29, 30), focused on urgency symptoms and urination frequency. Secondary outcomes included Quality of Life (QOL) scores (31) and subjective efficacy, evaluated at 2 weeks after the start of treatment, 4 weeks after the start of treatment, and at a 3 months follow-up. This methodical approach allowed for detailed analysis of treatment efficacy over time, with expansive details chronicled in Annex 1, 2, and 3.

Urine cytokine analysis: Urine samples were collected at the start and end of the four-week treatment to assess the biological effects of EA on OAB (BDNF, EGF, NGF, PGE2, MIP1β, and MCP1). Strict protocols minimized diurnal variations, and samples were analyzed via enzyme-linked immunosorbent assay (ELISA), correlating biochemical data with clinical results.

Safety and acceptance evaluation: Before each EA session, safety checks were performed to monitor for adverse events such as needle issues, dizziness, severe pain (VAS ≥ 8), and prolonged post-treatment discomfort (VAS ≥ 4). Additional metrics included monitoring for local hematoma, infections, or abscesses and documenting any secondary discomforts like fatigue and headache where VAS scores were ≥4 (32). Participant acceptance was rated on a five-point scale from “very difficult to accept” to “very easy to accept,” assessed after the first and sixth treatment sessions to gauge ongoing patient tolerance and satisfaction.

### 2.7 Statistical analysis

Intention-to-treat (ITT) analysis will be employed to account for all randomized participants, with missing data handled by carrying forward the last observation from the previous examination. Statistical analyses will be performed using SPSS version 25.0. For normally distributed or approximately normally distributed continuous data, results will be presented as mean ± standard deviation (SD). Comparisons between groups will be conducted using independent-samples *t*-tests. For skewed distribution data, results will be presented as median (interquartile range, IQR), and comparisons between groups will be performed using the Mann–Whitney U-test.

For repeated measurements from the same subjects, repeated-measures analysis of variance (ANOVA) will be used to evaluate differences across time points, between groups, and for interaction effects between time and group. Additionally, randomized block ANOVA with Dunnett’s method will be used to compare differences at multiple time points after treatment with baseline values.

## 3 Results

### 3.1 Patient demographics and baseline characteristics

**Figure 2:**
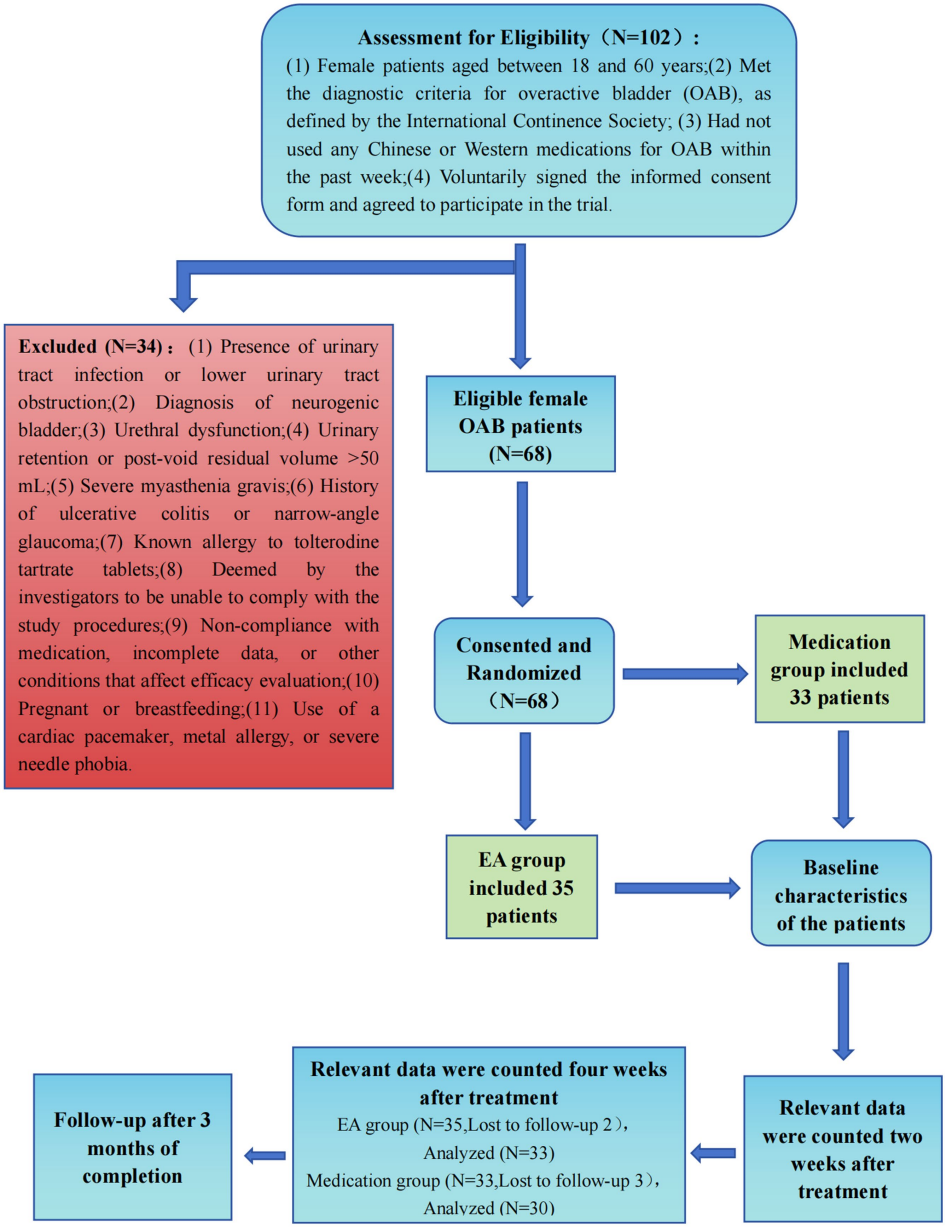
Consolidated standards of reporting trials profile. EA, Electroacupuncture.

This study enrolled 68 female OAB patients using a stratified randomization protocol, allocating 35 to the EA group and 33 to the medication group after excluding two severe cases to meet the sample cap (Figure 2). Despite slight discrepancies in severe case distribution, statistical blinding was maintained, identifying groups as “Group 1” and “Group 2” Statistical tests, including *t*-tests and chi-square tests, showed no significant differences between groups in age, disease duration, menopausal status, or disease severity, confirming comparable baseline characteristics. The EA group had an average age of 37.51 ± 12.61 years with a disease duration of 6.80 ± 2.06 months, while the medication group had an average age of 38.76 ± 10.19 years with a disease duration of 6.67 ± 2.41 months. Initial symptom scores and cytokine levels were also matched across groups, demonstrating balanced distribution at the outset. Dropout occurred with three in the medication group due to adverse reactions and two in the EA group due to work constraints shown in Table 1.

**Table 1:** Baseline characteristics.

Pre-treatment		EA group (*N* = 35)	Medication group (*N* = 33)	*P*
Age (years)		37.51 ± 12.61	38.76 ± 10.19	0.657
Disease duration (months)		6.80 ± 2.06	6.67 ± 2.41	0.806
Menopausal status (%)	Postmenopausal	7(20)	5(15.2)	0.600
	Not postmenopausal	28(80)	28(84.8)	
Disease severity (%)	Mild	17(48.6)	17(51.5)	0.872
	Moderate	11(31.4)	11(33.3)	
	Severe	7(20)	5(15.2)	
Urgency symptoms score		3(0)	3(1)	0.937
OABSS score		6(4)	5.5(3)	0.964
Quality of life score		5(0)	5(1)	0.592
BDNF(ng/mL)		2.97 ± 1.47	2.95 ± 1.61	0.950
EGF(ng/mL)		0.97 ± 0.21	0.96 ± 0.25	0.913
NGF(ng/mL)		11.14 ± 5.17	12.75 ± 6.43	0.258
PGE2(ng/mL)		3.81 ± 1.31	3.81 ± 0.93	0.999
MIP1β(pg/mL)		104.74 ± 33.43	112.18 ± 28.50	0.328
MCP 1(pg/mL)		94.43 ± 37.92	93.3 ± 40.03	0.905

### 3.2 Outcome measures analysis results

#### 3.2.1 Changes in scores following treatment

Two weeks post-treatment, the EA group exhibited significant QOL improvements compared to the medication group, as shown by a statistically significant Mann–Whitney *U*-test result (*p* = 0.002). No significant differences were observed in urgency symptoms or OABSS between the groups at this time (*p* > 0.05), emphasizing EA’s impact on QOL without affecting core symptoms. By 4 weeks, both treatments significantly improved urgency symptoms and QOL scores (*p* < 0.05), though OABSS changes remained statistically insignificant (*p* = 0.081), suggesting gradual therapeutic effects.

Detailed analysis within the EA group demonstrated marked improvements across all evaluated measures at both 2 and 4 weeks (*p* < 0.001), affirming EA’s efficacy in managing OAB and enhancing patient-reported outcomes. The medication group displayed similar improvements, especially at 4 weeks across most parameters, except for marginal significance in OABSS scores at 2 weeks (*p* = 0.052), indicating a delayed symptom reduction.

Three months after treatment, follow-up showed the EA group outperforming the medication group in urgency symptoms, OABSS, and QOL scores, substantiating EA’s long-term efficacy in OAB management and its potential as a sustainable treatment option (Table 2).

**Table 2:** Comparison of immediate OABSS, and subjective efficacy evaluation between two groups and their scores at 3 months follow-up.

Post-treatment		EA group (*N* = 35)	Medication group (*N* = 33)	*P*
Urgency symptoms score	2 weeks	1 (1)*	2 (2)*	0.173
	4 weeks	2 (1)*	2 (1)*	0.043
OABSS score	2 weeks	3 (3)*	4 (5)	0.291
	4 weeks	3 (3)*	4 (2)*	0.081
Quality of life score	2 weeks	2 (1)*	4 (2)*	0.002
	4 weeks	2 (1)*	3 (1)*	0.001
OABSS efficacy evaluation (4 weeks)	Marked effective	8 (22.9)	4 (12.1)	0.002
	Effective	23 (65.7)	12 (36.4)	
	Ineffective	4 (11.4)	17 (51.5)	
	Calculation of efficacy rate	31 (88.6)	16 (48.5)	
Subjective efficacy evaluation (2 weeks)	Very helpful	8 (24.2)	0 (0)	<0.001
	Moderately helpful	21 (63.6)	17 (56.7)	
	Slightly helpful	4 (12.1)	12 (40.0)	
	Not helpful at all	0 (0)	1 (3.3)	
Subjective efficacy evaluation (4 weeks)	Very helpful	23 (69.7)	10 (33.3)	0.002
	Moderately helpful	9 (27.3)	10 (33.3)	
	Slightly helpful	1 (3.0)	10 (33.3)	
	Not helpful at all	0 (0)	0 (0)	
Three months follow-up after treatment completion	Urgency symptom score	2.30 ± 0.68	2.86 ± 0.81	0.004
	OABSS score	3.75 ± 2.16	5.06 ± 2.09	0.018
	Quality of life score	2.45 ± 0.79	2.96 ± 1.06	0.034

* represents *p* < 0.001 compared with before treatment. The description of efficacy evaluation is presented as the number of cases (%).

#### 3.2.2 Evaluation of EA efficacy

Our study demonstrated a clear statistical advantage of EA over medication in treating OAB. A chi-square test revealed a significant difference in treatment efficacy between groups (*p* = 0.002), with 88.6% of patients in the EA group showing marked improvement compared to 48.5% in the medication group. Subjective efficacy assessments from 63 participants revealed significant enhancements in patient-reported outcomes at both 2-week and 4-week intervals post-treatment (*p* < 0.05), as detailed in Table 2, confirming EA’s superior effectiveness in symptom relief and enhancing overall well-being, advocating for its broader application in OAB treatment.

#### 3.2.3 Urinary cytokines analysis

Our analysis of urinary cytokines in 63 participants using independent *t*-tests found no significant differences in post-treatment concentrations of BDNF, EGF, NGF, PGE2, MIP1β, and MCP1 between the EA and medication groups, suggesting similar biochemical impacts. Both treatments led to significant reductions in BDNF levels (EA: *p* < 0.001, medication: *p* = 0.038), pointing to a common physiological effect. No other cytokines showed significant changes, underscoring BDNF’s specific modulation. A further comparison of pre- and post-treatment cytokine levels confirmed these findings, reinforcing the comparable efficacy of both treatments (Table 3). Notably, in patients with severe OAB, significant changes in all measured cytokines post-treatment highlighted both therapies’ potential to affect inflammatory and neurotrophic pathways in more advanced cases (Table 4).

**Table 3:** Comparison of urinary cytokine levels before and after treatment.

Urinary cytokine		Difference (x ± s)	Pair *t*-values	*P*
EA group(*N* = 33) (ng/mL)	BDNF	−2.25 ± 2.62	−4.928	<0.001
	EGF	−0.01 ± 0.36	−0.102	0.919
	NGF	−1.86 ± 7.64	−1.397	0.172
	PGE2	−0.15 ± 1.61	−0.556	0.582
	MIP1β	−1.51 ± 35.95	−0.242	0.811
	MCP1	−9.85 ± 67.06	−0.844	0.405
Medication group(*N* = 30) (ng/mL)	BDNF	−1.08 ± 2.73	−2.171	0.038
	EGF	0.096 ± 0.39	1.338	0.191
	NGF	1.49 ± 7.73	1.059	0.298
	PGE2	0.10 ± 1.29	0.432	0.669
	MIP1β	12.65 ± 38.89	1.782	0.085
	MCP1	−20.99 ± 64.75	−1.775	0.086

**Table 4:** Comparison of urinary cytokine levels before and after treatment in severe female OAB patients.

Urinary cytokine		Pre-treatment (*N* = 12)	Post-treatment (*N* = 11)	*P*
Severe female OAB patients (EA group and medication group, *N* = 12) (ng/mL)	BDNF	4.46 ± 2.40	2.11 ± 0.59	0.013
	EGF	1.58 ± 0.25	1.00 ± 0.19	<0.001
	NGF	8.92 ± 2.42	4.26 ± 1.08	<0.001
	PGE2	4.59 ± 0.98	2.10 ± 0.72	<0.001
	MIP1β	126.75 ± 25.79	89.53 ± 13.82	0.007
	MCP1	71.03 ± 16.17	63.39 ± 26.72	0.468

#### 3.2.4 Safety and acceptance evaluation

Safety and acceptance of EA were thoroughly evaluated among 35 participants, with minimal discomfort reported; 73.5% had a VAS pain score of 1, and 26.5% scored 2. Additionally, acceptance was high, with 82.4% finding EA very easy to accept. The absence of serious complications or significant discomfort during or after sessions underscores EA’s safety and tolerability, supporting its use as an effective treatment for overactive bladder. These results are further detailed in subsequent sections of the study.

## 4 Discussion

This randomized controlled trial revealed that EA significantly enhances quality of life and mitigates urgency symptoms in female patients with OAB, demonstrating comparable efficacy to conventional medication. Notably, our findings highlight the potential of EA as a safe, well-tolerated, and effective treatment modality, with a higher overall effective rate (88.6%) compared to medication (48.5%). The analysis of urinary cytokines post-treatment, particularly the significant changes in BDNF levels within the EA group, supports the therapeutic role of EA in modulating neural pathways involved in OAB.

The theoretical underpinnings of our research, supported by existing literature, suggest that normal micturition relies on the integrity of neural pathways (33), which can be modulated by therapies like EA. EA stimulation of the bilateral BL33 (S3, posterior sacral foramen) effectively targets the S3 nerve, known for its robust innervation of detrusor muscles. The sacral three nerve roots emerge as pivotal neural pathways for stimulating the vesicourethral muscle, making S3 the primary acupoint choice. Another selected acupoint in the study is SP6, positioned directly above the tip of the medial malleolus and posterior to the tibia according to international standards for localization. The cutaneous distribution of the iliohypogastric nerve of the fourth lumbar spinal segment encompasses this acupoint, with the tibial nerve traversing beneath the SP6 site. From a neuroanatomical perspective, the application of stainless steel needle electrodes above the medial malleolus for electrical stimulation of the tibial nerve aids in achieving direct sensory and motor control of the bladder and pelvic floor. This indirect approach contributes to the overall therapeutic objectives. However, the challenge lies in accurate localization, impeding widespread clinical application. Electroacupuncture sensation alone is insufficient for judging BL33 localization and entry accuracy due to the deep location of BL33 and the physiological curvature of the caudal vertebrae. Addressing this, the study combines ancient localization methods with modern medical sacral nerve stimulators, enhancing the precision of BL33 acupuncture targeting the S3 nerve. Considering the distinct responses of different sacral nerves to EA stimulation further enhances the objective determination of needle accuracy, ensuring precise needle placement. Our findings provide empirical support for this, with EA’s targeted stimulation of BL33 and SP6 potentially modulating the neural pathways involved in OAB.

Currently, the diagnosis of OAB is based primarily on patient-reported symptoms, with confirmatory tests used to exclude other conditions, reflecting a lack of objective, measurable diagnostic markers (34). Recent studies suggest that specific urinary cytokines are closely associated with urgency and frequency symptoms, and may serve as potential biomarkers for OAB. Key cytokines include NGF, BDNF, MCP-1, PGE2, EGF, and MIP-1β (35–38). Elevated urinary levels of NGF and BDNF have been linked to involuntary detrusor contractions via receptor pathways, contributing to urgency, frequency, and incontinence. Clinical trials have reported a significant increase in NGF in OAB patients, positively correlated with symptom severity, while treatment with anticholinergics reduces NGF levels, supporting its relevance to disease activity (39). BDNF, essential for neuronal remodeling, has also been implicated in OAB through its effects on sensory nerve hyperexcitability and dysfunctional signaling (40, 41), underscore the biological impact of EA treatment and suggest avenues for further exploration. PGE2 is involved in non-cholinergic detrusor contraction and is elevated in idiopathic OAB patients, with levels correlating with symptom severity (42). EGF, recognized for its urothelial regulatory role, is also increased in the urine of patients with chronic interstitial cystitis (IC) (43). Notably, patients with mild to moderate IC often present with only urgency and frequency (44–46), suggesting possible overlap with OAB. Furthermore, EGF, MIP-1β, and MCP-1 may contribute to voiding symptoms in IC and could also be relevant to OAB pathogenesis (36, 47, 48). Despite no significant differences in cytokine levels between treatment groups post-treatment, the within-group changes in BDNF levels and the clinical improvement in severe OAB patients post-treatment highlight the potential of these markers in understanding OAB’s pathophysiology and treatment response. This points to the necessity for further research into the mechanisms of EA in OAB management and its role in neural modulation.

The strengths of our investigation lie in its rigorous randomized controlled design, the innovative application of EA based on acupuncture principles and neural modulation, and the comprehensive evaluation of clinical outcomes and urinary cytokines as biological markers. Our methodological rigor, including randomization and statistical blinding, enhances the credibility of the findings, providing a robust framework for comparing the efficacy, safety, and patient acceptance of EA versus medication. However, the study acknowledges certain limitations. The focus on a female patient cohort limits the generalizability of our results to broader populations, including male patients. Additionally, despite efforts to maintain statistical blinding, the inherent differences in treatment modalities could introduce biases. Future research should aim to overcome these limitations, exploring larger and more diverse patient populations to validate and broaden our findings. Further investigation is needed to delineate specific patient profiles that may derive the most benefit from EA versus medication, enhancing the precision and personalization of OAB treatment strategies.

## 5 Conclusion

The study substantiates electroacupuncture’s efficacy and safety in managing female OAB, offering a valuable addition to the existing therapeutic modalities. Future research should aim to expand on these findings, exploring the long-term effects of EA, its applicability in diverse patient populations, and further elucidation of its underlying mechanisms through urinary cytokine analysis. The exploration of EA’s role in neural modulation opens new avenues for non-pharmacological OAB treatment, advocating for its integration into clinical practice.

## Data Availability

The datasets presented in this study can be found in online repositories. The names of the repository/repositories and accession number(s) can be found in the article/Supplementary material.
