# Supplementary material for: Effectiveness and safety of electroacupuncture in female overactive bladder: a randomized controlled trial investigating sacral and tibial nerve modulation
Source: Front Med (Lausanne). 2025 Sep 16;12:1579276. doi: 10.3389/fmed.2025.1579276 (PMC12479545; doi:10.3389/fmed.2025.1579276)
Supplement: Supplementary file 2 [file Table_2.DOC]

**Annex 1**. Overactive Bladder Symptom Score（OABSS）Scale

| Question | Symptom | Frequency times | Scores (please tick "√" in this column) |
| --- | --- | --- | --- |
| 1  Number of times you urinate during the day | How often do you urinate from waking up in the morning to falling asleep at night? | ≤7 | 0 |
|  |  | 8-14 | 1 |
|  |  | ≥15 | 2 |
| 2  Number of times you urinate during the night | How many times do you get up because of peeing in the time you fall asleep at night to when you wake up in the morning? | 0 | 0 |
|  |  | 1 | 1 |
|  |  | 2 | 2 |
|  |  | ≥3 | 3 |
| 3  Urinary urgency | Is there a sudden urge to urinate that is unbearable at the same time? | NOT | 0 |
|  |  | weekly＜1 | 1 |
|  |  | weekly≥1 | 2 |
|  |  | daily＝1 | 3 |
|  |  | daily2-4 | 4 |
|  |  | daily≥5 | 5 |
| 4  Urge incontinence conditions | Do you suddenly want to urinate and at the same time can't stand it and have urinary incontinence? | NOT | 0 |
|  |  | weekly＜1 | 1 |
|  |  | weekly≥1 | 2 |
|  |  | daily＝1 | 3 |
|  |  | daily2-4 | 4 |
|  |  | daily≥5 | 5 |
| Total Score: | | | |

(Markedly effective: OABSS score reduction greater than 70% at the end of the treatment course; Effective: OABSS score reduction between 30%-70% at the end of the treatment course; Ineffective: OABSS score reduction less than 30% at the end of the treatment course. Efficacy rate (%) = (Markedly effective + Effective) / Total number of participants × 100%.)

**Annex 2**. Quality of Life Index (QOL) score scale

| If the symptoms of urinary frequency and urgency affect your life, how do you rate the quality of life at this stage? | | | | | | | |
| --- | --- | --- | --- | --- | --- | --- | --- |
| level of satisfaction | Happy | satisfied | generally satisfied | with little impact | Not very satisfied | distressed | very bad |
| Scores (please tick "√" in this column) | 0 | 1 | 2 | 3 | 4 | 5 | 6 |

**Annex 3**. Subjective patient efficacy evaluation: patients were asked whether the treatment therapy helped to control the symptoms of urinary frequency and urgency using a 4-point scale.

| ■ Not helpful at all- Level 0 | ■ Slightly helpful - level 1 |
| --- | --- |
| ■Moderately helpful - Level 2 | ■ Very helpful - 3 levels |
