# Supplementary material for: Effectiveness and safety of electroacupuncture in female overactive bladder: a randomized controlled trial investigating sacral and tibial nerve modulation
Source: Front Med (Lausanne). 2025 Sep 16;12:1579276. doi: 10.3389/fmed.2025.1579276 (PMC12479545; doi:10.3389/fmed.2025.1579276)

Chinese Clinical Trial Registry (ChiCTR) was established in 2005 by professor Wu Taixiang and Li Youping team, West China Hospital, Sichuan University, and the Ministry of Health of China assigned it to be the representative of China to join WHO ICTRP in 2007（**http://www.chictr.org.cn**）.The Chinese Clinical Trial Registry is a non-profit organisation. Chinese Clinical Trial Registry provides the services include register for trials, consultation for trial design, central randomization for an allocation sequence, peer review for draft articles and training for peer reviewers. CCRB Clinical Trials Registry, CUHK, and the Acupuncture-Moxibustion Clinical Trial Registry in China Academy of Chinese Medicine are the Partner Registry of Chinese Clinical Trial Registry. Our trial is registered with the Chinese Clinical Trial Registry (Registration number: ChiCTR1900021372).


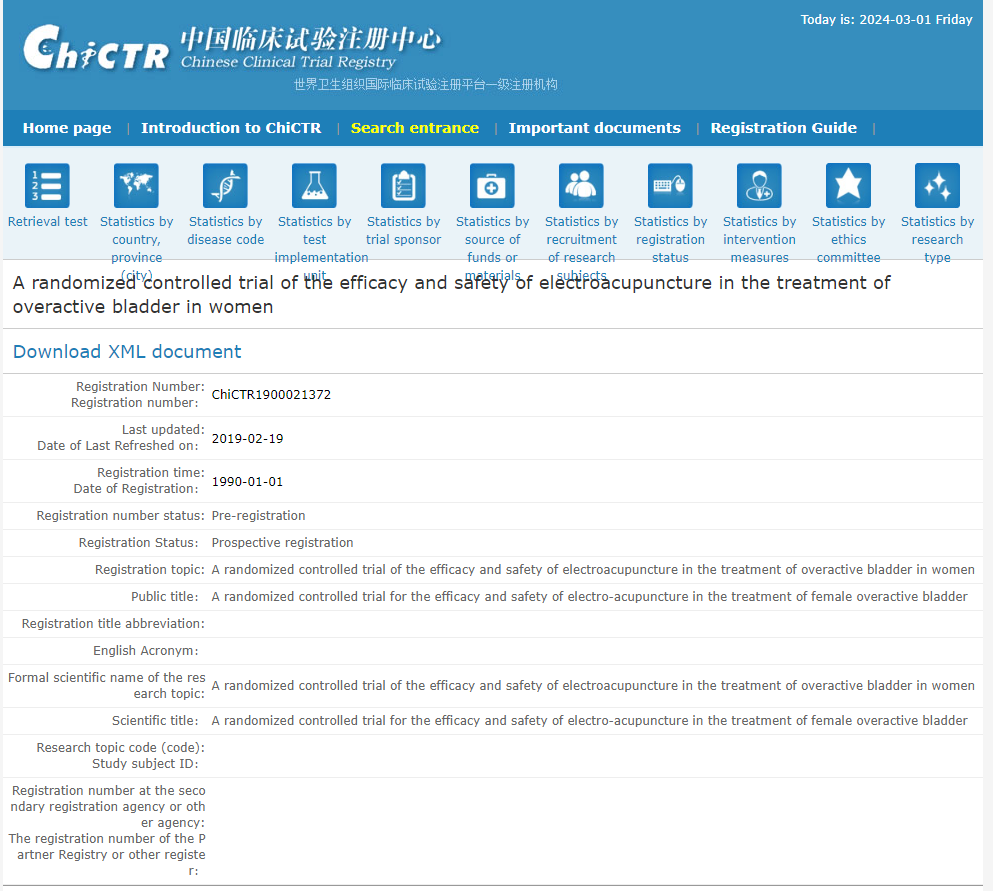

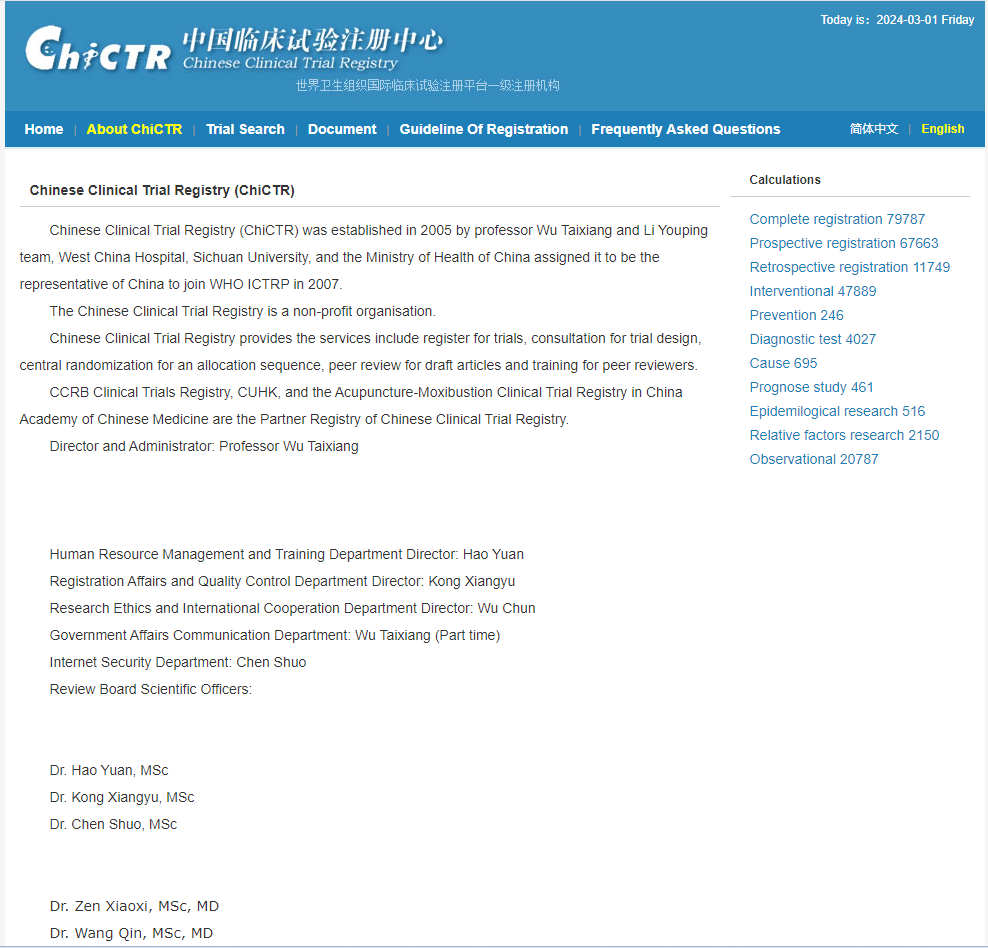

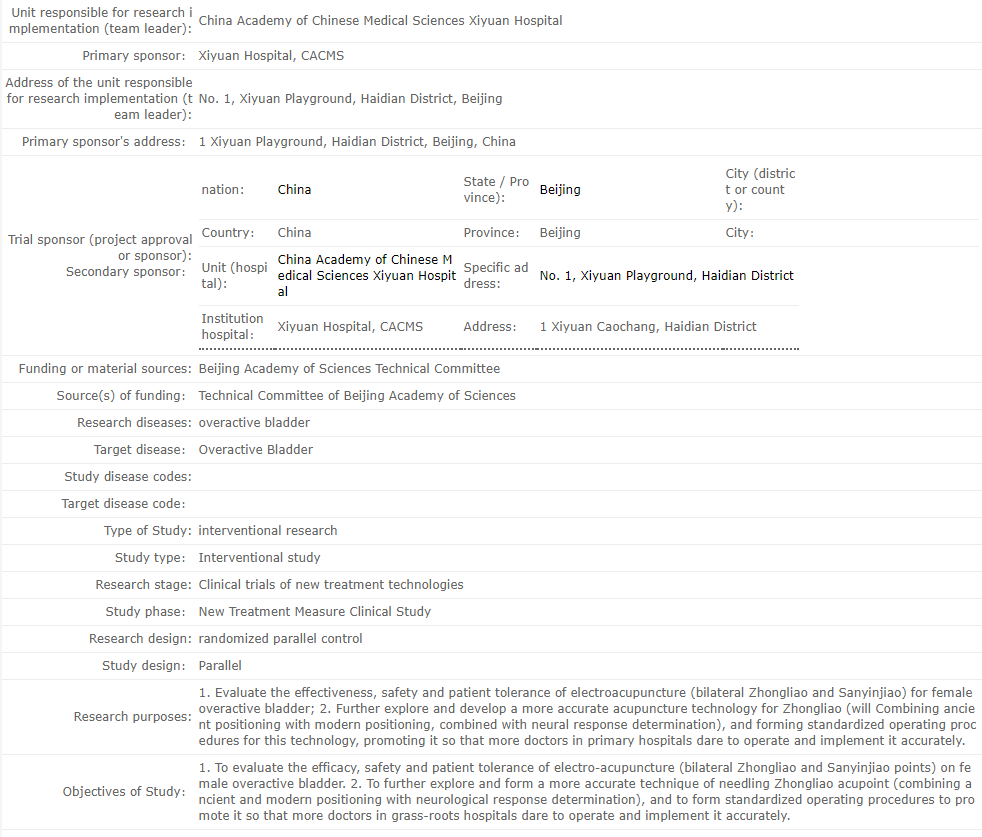

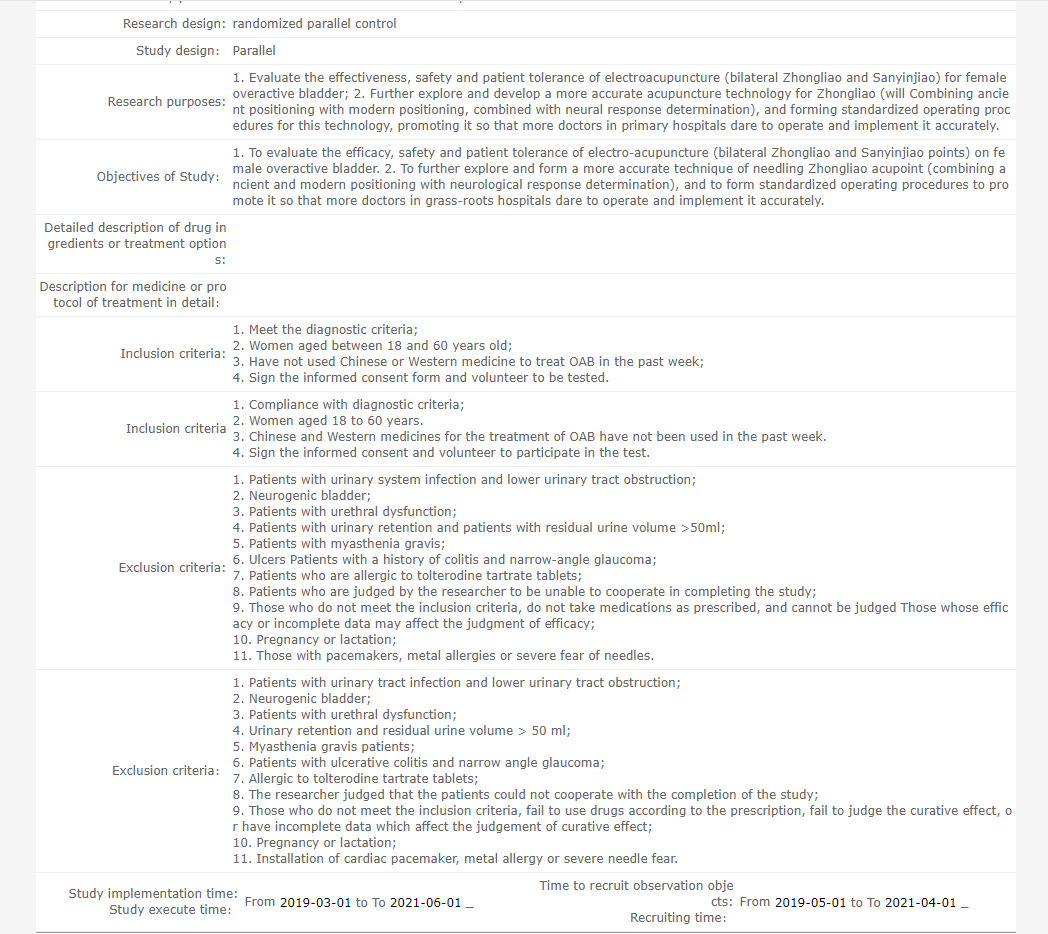

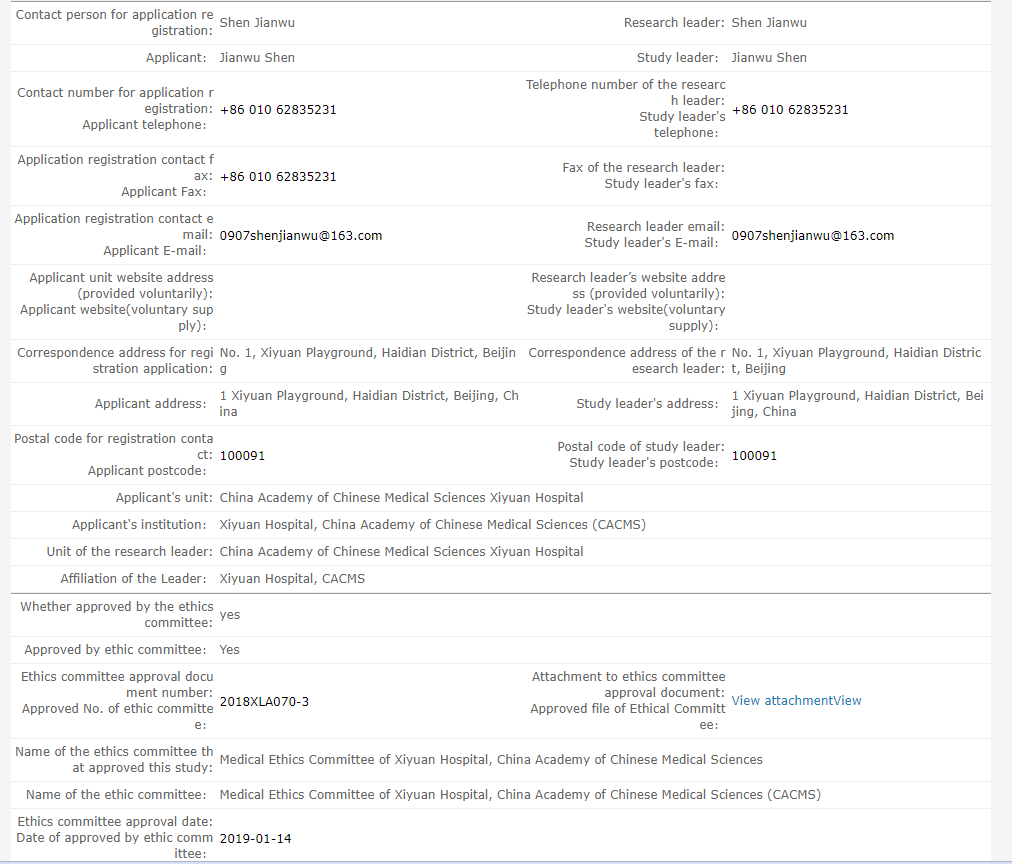

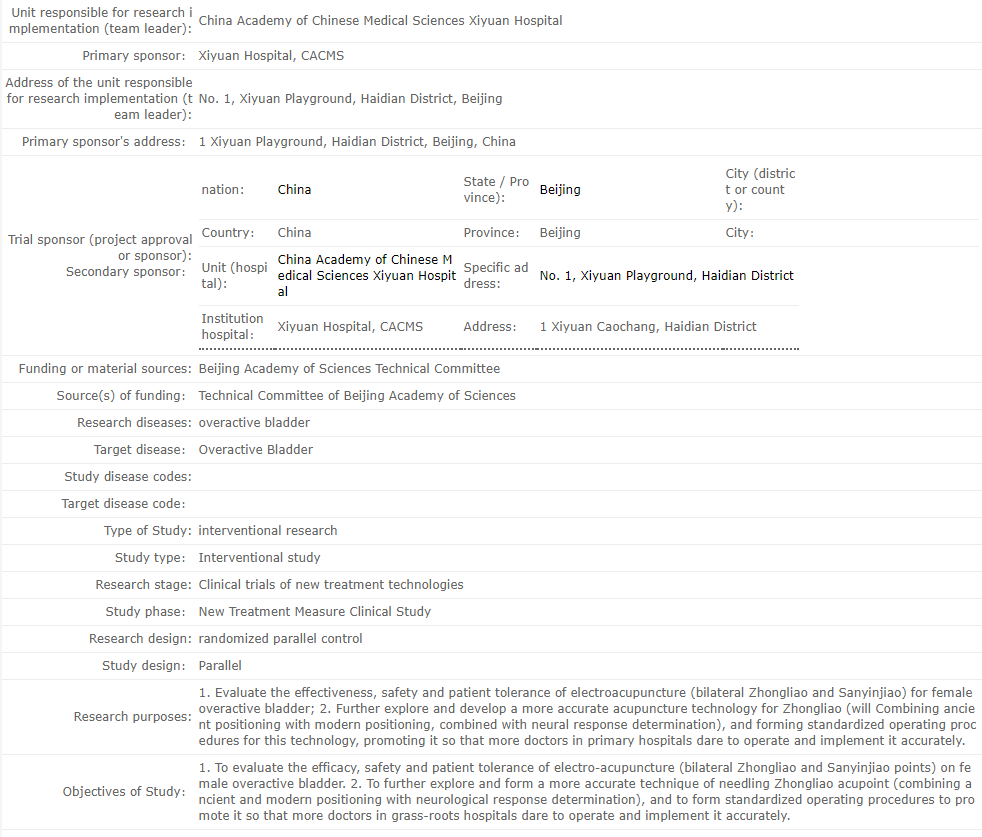

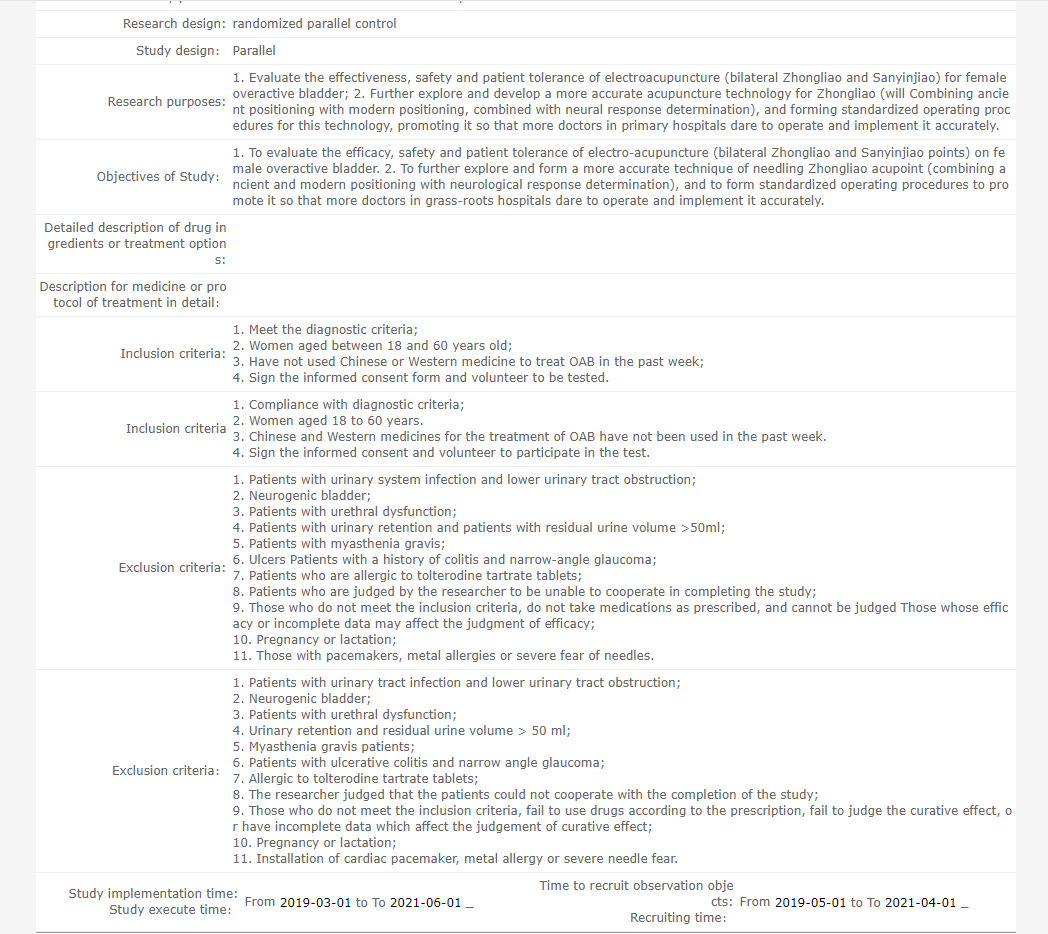

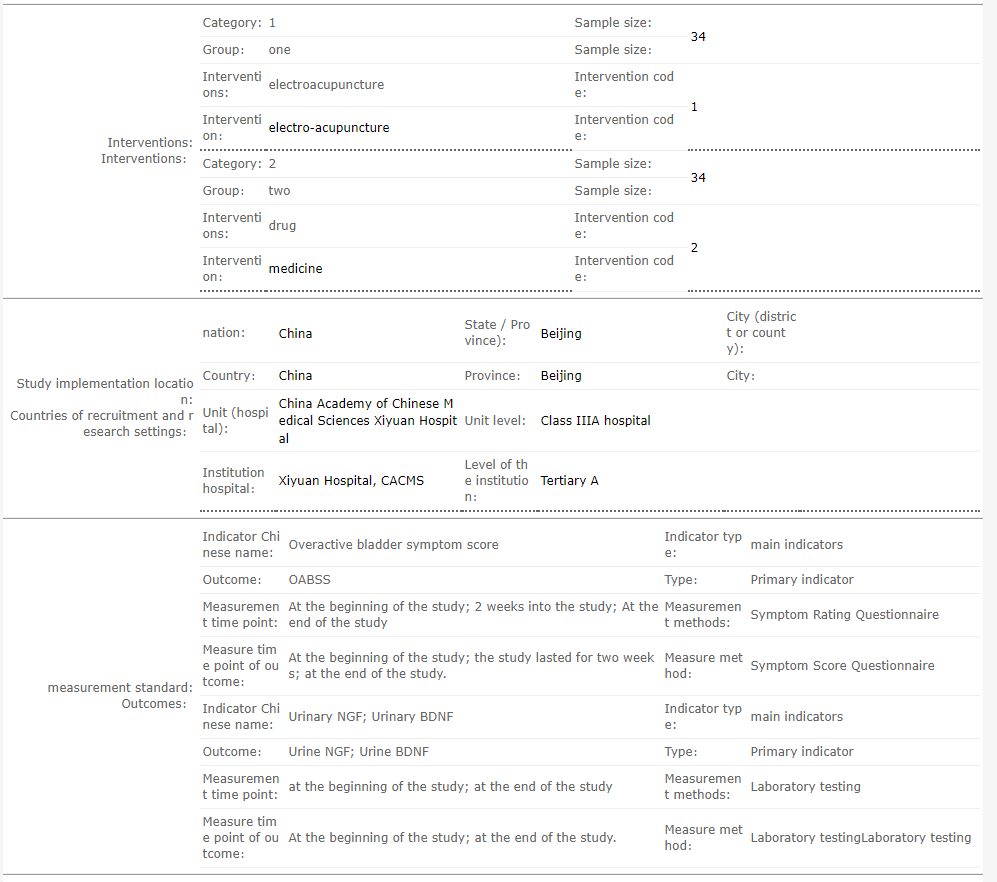

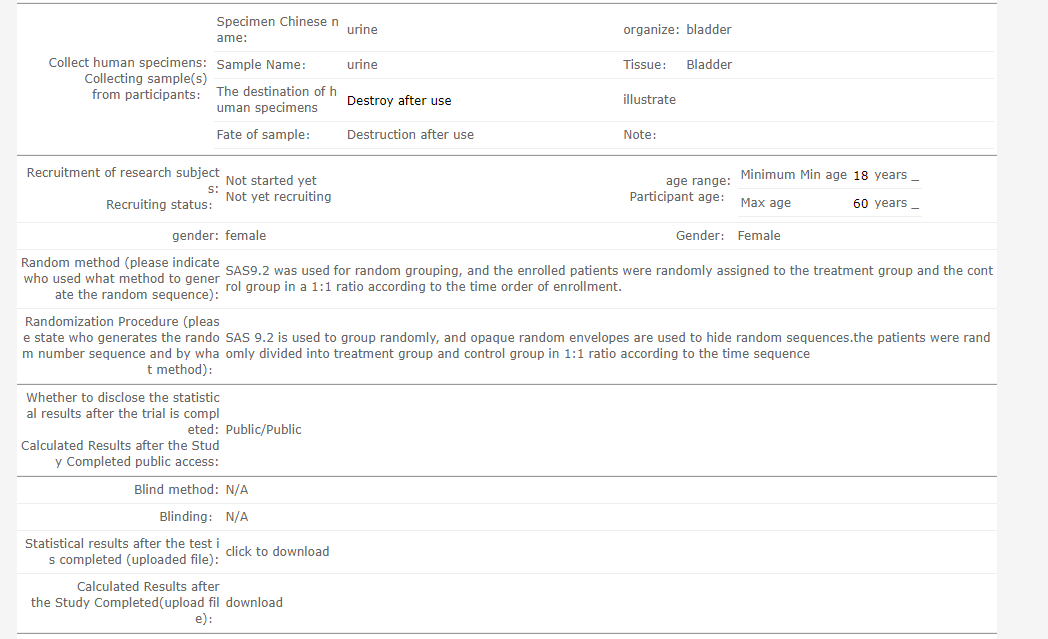

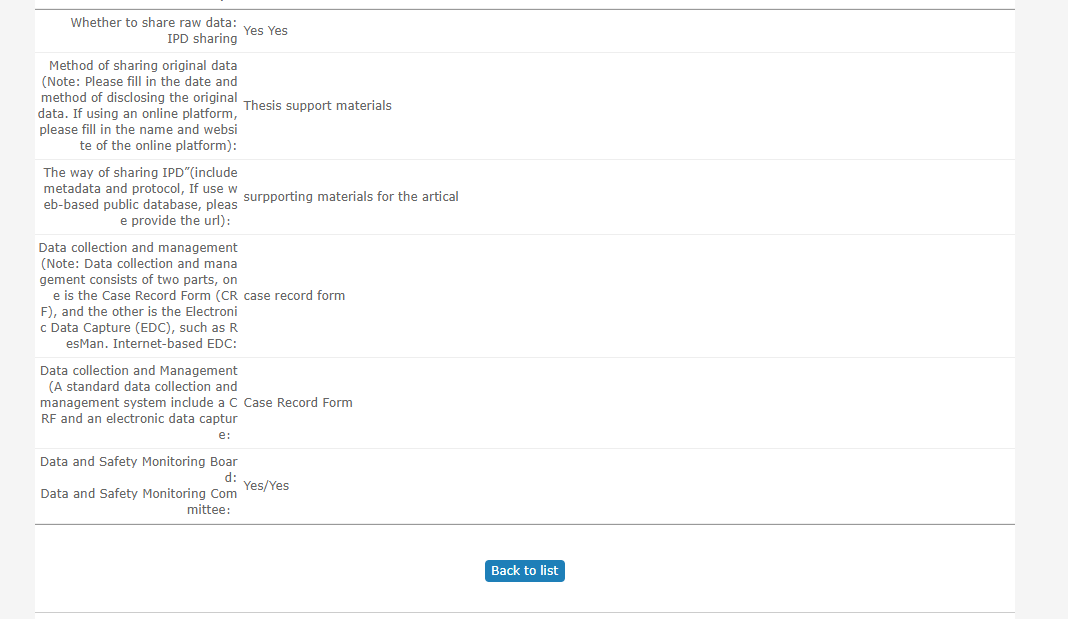

Supplement: Supplementary file 4 [file Table_4.DOCX]
